# Atomic Diffusion within Individual Gold Nanocrystal

**DOI:** 10.1038/srep06765

**Published:** 2014-10-24

**Authors:** Gang Xiong, Jesse N. Clark, Chris Nicklin, Jonathan Rawle, Ian K. Robinson

**Affiliations:** 1 London Centre for Nanotechnology, University College London, London WC1H 0AH, UK; 2 Diamond Light Source, Harwell Campus, Didcot, OX11 0DE, UK; 3 Research Complex at Harwell, Harwell Oxford, Didcot, OX11 0FA, UK

## Abstract

Due to their excess surface free energy and structural instabilities, nanoparticles exhibit interesting physical and chemical properties. There has been an ever-growing interest in investigating these properties, driven by the desire to further miniaturize electronic devices, develop new functional materials and catalysts. Here, the intriguing question of how diffusion evolves in a single nanoparticle is investigated by measuring the spatial and temporal variations of the diffracted coherent X-ray intensity during copper diffusion into a gold nanocrystal. Dislocation loops formed from the insertion of single layer of extra atoms between neighbouring gold host lattice planes are detected. Au-Cu alloy channels are found to penetrate the nanocrystal due to the differential diffusion rate along different directions. With the advent of higher brilliance sources and free-electron-lasers, Bragg Coherent X-ray Diffraction Imaging can play an important role in unveiling atomic behaviours in three dimensions for nanomaterials during various fundamental processes.

Diffusion is a transport phenomenon whereby the random thermally-activated movement of atoms results in their net transport and intermixing without the bulk motion^1^. How diffusion happens on the atomic scale in a solid is a fundamental issue and often the crucial step in many processes such as doping silicon wafers to make semiconductor devices^2^,^3^, transport of thermal neutrons in nuclear power reactors^4^ and permeation of atoms through membranes^5^,^6^. Rates of numerous chemical reactions are dependent on how fast diffusion can bring reactants together or deliver them to reaction sites on enzymes or other catalysts^7^,^8^,^9^. Accurate models and understanding of these diffusion mechanisms will aid the ability to control the synthesis of materials with desired properties, and to design and fabricate devices with improved performance^10^,^11^,^12^,^13^. In particular, as functional materials and devices with reduced dimensions have been receiving great attention in recent years, it is imperative to develop techniques suited for performing diffusion characterization at the nano- and micro-scale level^14^,^15^,^16^,^17^.

Conventional methods such as mechanical and sputter profiling^18^, secondary ion mass spectrometry^19^,^20^ and electron microprobe analysis^21^,^22^, investigate diffusion inside solids by measuring the profile of the diffusing element in a solid material and yield a macroscopic quantity-the diffusion coefficient. On the microscopic level, however, it is difficult to directly observe the relatively slow process of the diffusion of atoms in solids because the timescales are too long for experimental techniques that depend on high energy resolution, such as inelastic scattering^23^. Quasi-elastic neutron scattering^24^,^25^ and Mössbauer spectroscopy^26^,^27^ can address this issue only for a small number of isotopes and very fast diffusivities. These conventional methods have been applied to investigate diffusion behaviours for samples of nanoparticle ensembles, to obtain the diffusion coefficient^17^,^28^,^29^,^30^. X-ray photon correlation spectroscopy^31^,^32^ based on the diffuse scattering of coherent X-rays has recently been used to elucidate the dynamic behaviour of atoms as a function of their neighbourhood. In these work, it is necessary to integrate and average the signals of a number of structures or from a relatively large area, and sample heterogeneity can cause ambiguity in the analysis results. Transmission electron microscopy (TEM) can resolve the behaviour of individual nanoparticle^33^,^34^,^35^. However, for TEM the the destructive thinning used in sample preparation can be a severe limitation. Also for diffusion studies, the illumination of the nano-structured sample by the electron beam can completely alter the diffusion behaviour^33^. To date, investigating the diffusion properties for individual nanoparticle with a three-dimension capability is still difficult.

Here we demonstrate a successful investigation of the three-dimensional diffusion behaviours at the atomic level in a single gold nanocrystal with copper as the diffusing species, using Bragg Coherent X-ray Diffraction Imaging^36^,^37^,^38^,^39^ (BCDI) in an ultrahigh vacuum environment. BCDI recovers the complex electron density of the samples with picometre sensitivity and it is able to detect very small lattice distortion within nanocrystals. Indeed, the 'picometre sensitivity' refers to lattice displacement which is much finer than the atomic spacing. The spatial resolution of the retrieved image itself is around 20 nm in these experiments^37^,^39^. The ability to achieve picometre sensitivity from a lower resolution measurement is due to the fact that the strain fields are long-ranged, therefore atomic level defects, such as lattice distortion and dislocation loops, can be identified by their strain signatures. BCDI can currently examine nanocrystals as small as a few tens of nanometers, limited by the diffracted signal obtained over a reasonable data collection time. This will be improved with the new radiation source, i.e. free-electron-laser, becoming available and the developments in beamline optics. In BCDI, the recovered electron density comprises the amplitude, corresponding to the crystal morphology, and the phase, which is related to the displacement field of the atoms from the ideal crystal lattice. The choice of Bragg peak determines the direction in which the lattice distortions can be visualised. Results obtained from non-coplanar Bragg peaks can be combined to recover the full strain tensor^38^. In this study, experiments were performed in an ultrahigh vacuum chamber with a base pressure of 5 × 10^−9^ mbar, at beamline I07 of the Diamond Light Source. Time dependent diffraction patterns were recorded by rocking the gold nanocrystal around its (111) Bragg diffraction peak, during copper diffusion at 300°C, and collated to form three-dimensional data sets. By performing reconstruction from the diffraction data using a guided approach^40^ and partial coherent correction^41^, three-dimensional images of the gold nanocrystal, including both the phase and amplitude, at different times of diffusion, were obtained and analysed to reveal the evolution of the diffusion of copper.

## Results

**Figure 1:**
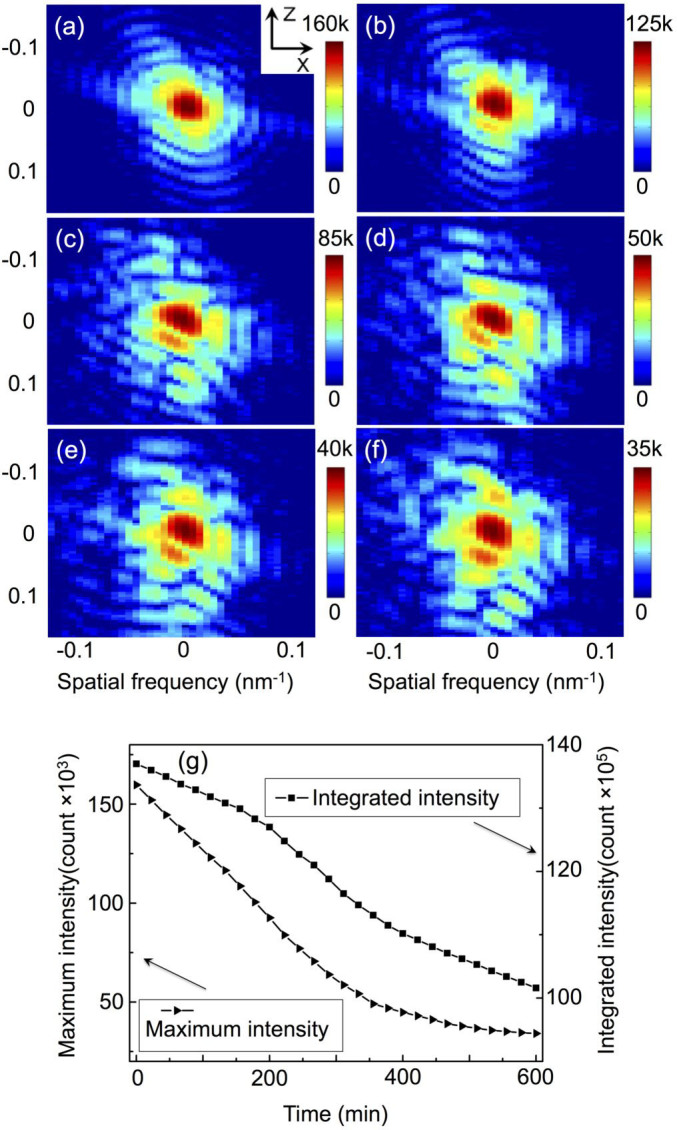
The patterns at the centre position of the three dimensional rocking curve of the gold (111) diffraction at the copper diffusion time of 0 hr (a), 2 hrs (b), 4 hrs (c), 6 hrs (d), 8 hrs (e), and 10 hrs (f), with a XZ plane view; the time dependence of the maximum diffraction intensity and the integrated diffraction intensity from the BCDI measurements (g).

Figures 1a–f show the patterns, for the gold (111) diffraction at the copper diffusion time of 0, 2, 4, 6, 8, and 10 hrs, respectively, as a cut-off XZ plane view at the centre position of the three dimensional diffraction rocking curve (All the cut-off images presented in this paper are XZ views unless stated otherwise.). One can see that before copper diffusion the diffraction patterns show the modulated fringes resulting from the coherent illumination and the finite size of the nanocrystal. The fringes present a fairly symmetric pattern, and are most prominent in the facetted directions of the nanocrystal. After 4 hours of diffusion, the diffraction patterns (Fig. 1c–f) become asymmetric with distorted fringes, which is due to the inhomogeneous lattice distortions resulting from the copper atoms diffused into the gold lattice. The phase diagram of Au-Cu system^42^ predicts that at 300°C, there are three stable Au-Cu alloys, CuAu_3_, CuAu and Cu_3_Au. In our experiment, after 10 hours of Cu diffusion, we find clear powder ring traces for Cu_3_Au, indicating that one of the Au-Cu alloys was formed due to the copper diffusion into the gold host lattice. The scale bars of Fig. 1a–f show that the maximum intensity of the diffraction pattern gradually decreases as the diffusion proceeds. This trend can be seen more clearly in Fig. 1g, which presents the time dependence of the maximum diffraction intensity and the integrated diffraction intensity, and both of them decrease monotonously. The diffraction intensity decrease is due to the diffusion-related decrease of the gold nanocrystal volume, with some gold crystalline parts gradually being turned into Au-Cu alloy.

**Figure 2:**
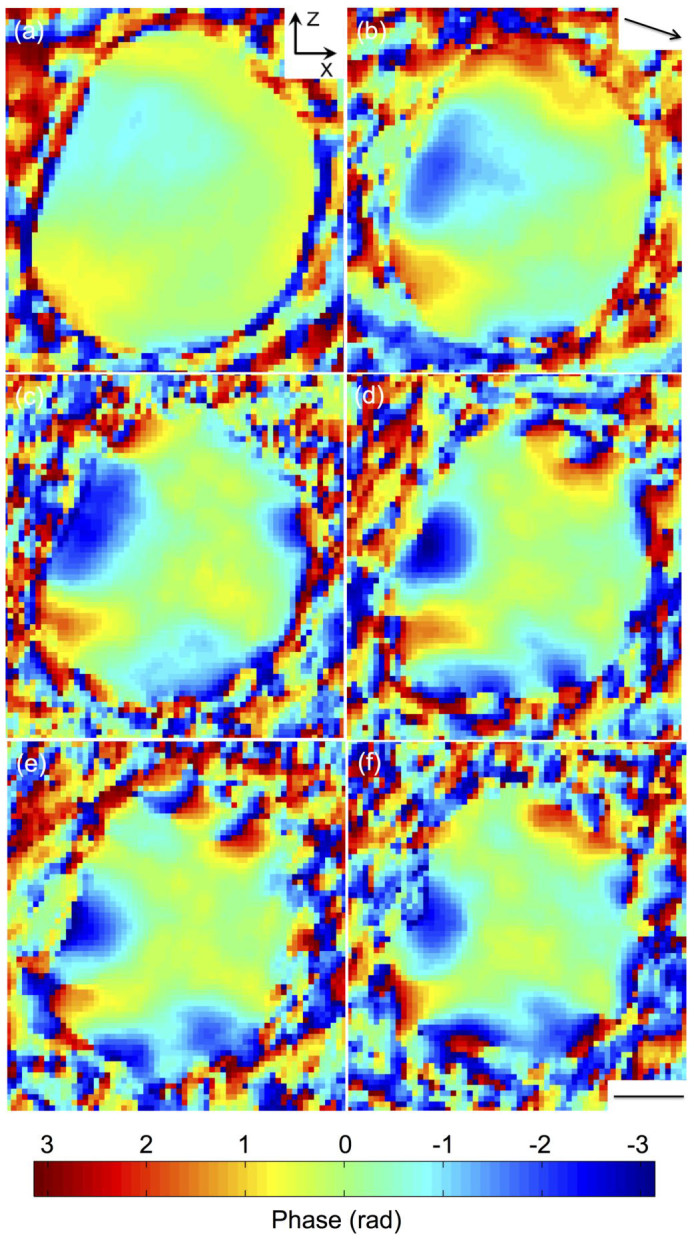
The reconstructed phase images, as cut-off view at the centre of the gold nanocrystal at diffusion time of 0 hr (a), 2 hrs (b), 4 hrs (c), 6 hrs (d), 8 hrs (e), and 10 hrs (f). Note that the phase becomes very noisy outside the crystal, where the amplitude of the complex density drops to zero. Arrow in (b) shows the Q-vector direction. Scale bar = 100 nm.

Figures 2a–f are the reconstructed phase images, as cut-off views at the centre of the gold nanocrystal at diffusion times of 0, 2, 4, 6, 8, and 10 hrs, respectively. The phase is a projection of the lattice distortions onto the (111) Q vector (shown as the arrow in Fig. 2b). It can be seen that before diffusion, the gold nanocrystal is almost strain free inside, with small phases in the range of ±0.5 rad (here a phase of 2π represents a displacement equal to 0.235 nm, the gold lattice spacing in the {111} direction). These phase features are located at the near surface regions where two facets meet, which we refer to as the crystal ‘edges'. The ‘edges' are curved regions on the equilibrium crystal shape^43^ where steps tend to be in motion and fluctuating^44^, and the weak phase features near the ‘edges' are due to strains generally associated with the shape of nanoparticles^45^. With diffusion proceeding, strain increases significantly inside the nanocrystal. After 6 hrs diffusion (Fig. 2d), there are three characteristic dipole-shaped phase structures, two near the top and one near the left bottom of the crystal. Each of them shows a clear phase wrap where the phase changes abruptly from -π to π over a small volume, indicating atom displacements are equal to the lattice spacing. These are attributed to dislocation loops^46^,^47^, consisting of edge dislocations formed from the insertion of a single plane of atoms over a small region between two adjacent lattice planes, and the relaxation of the strain field surrounding it. Dislocation loops have recently been identified in silicon nanowires upon mechanical bending, where the extra plane consists of strained silicon atoms^48^. In the case here, the extra plane of atoms may consist of either gold or copper, or the mixture of the two. The phase agrees well with model calculations of dislocation loop as illustrated in Supplementary Information 2. It can also be seen from Fig. 2 that the dislocation loops are formed near the crystal ‘edges' where facets meet. These are apparently the locations where defects are likely to be generated, corroborated by the observation noted above that there were already some shape-induced strains at these positions before diffusion. This means it is easier for the diffusion to proceed at positions where strains (defects) are already present.

**Figure 3:**
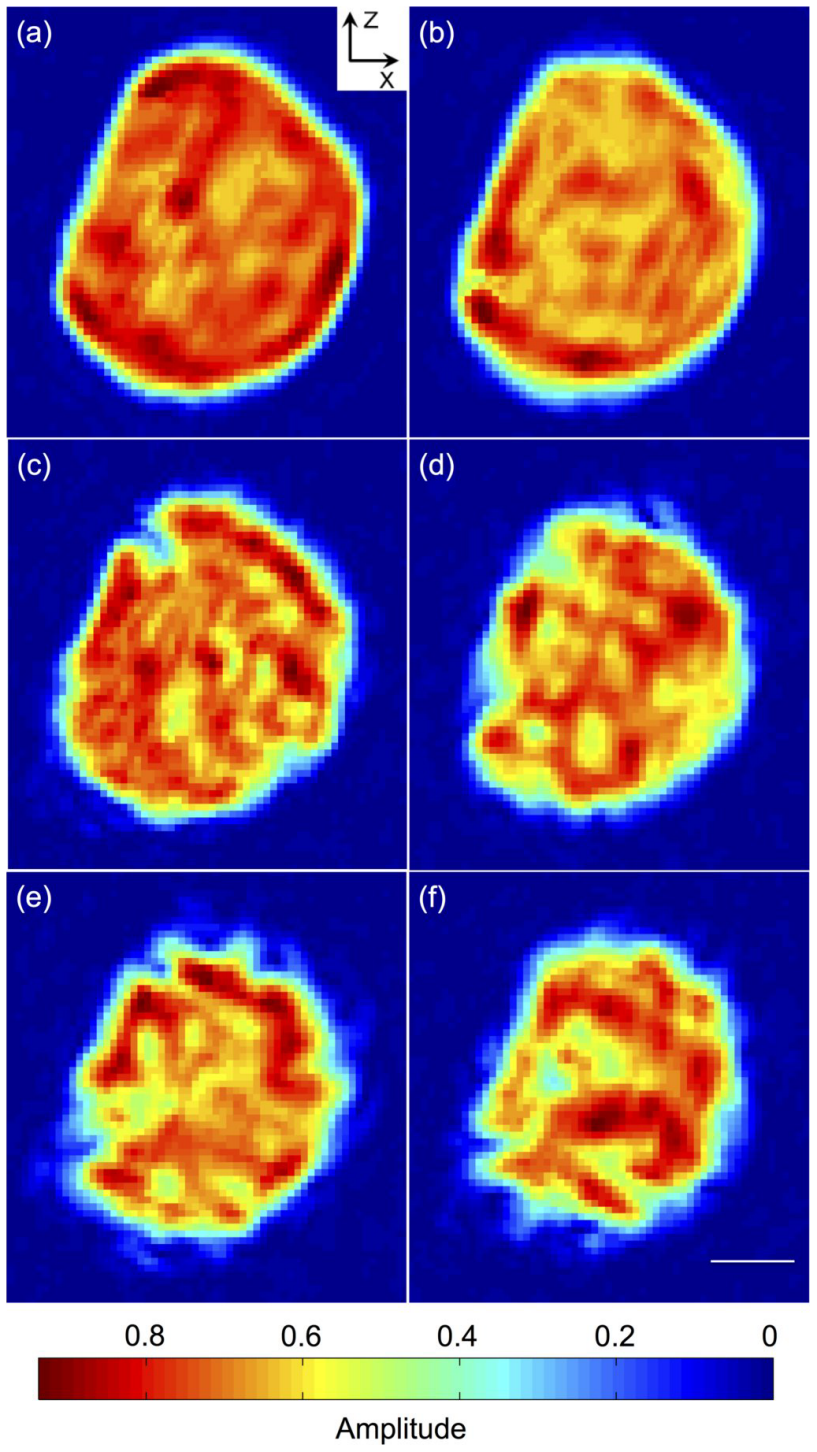
The reconstructed amplitude images, as cut-off view at the centre of the gold nanocrystal at diffusion time of 0 hr (a), 2 hrs (b), 4 hrs (c), 6 hrs (d), 8 hrs (e), and 10 hrs (f). Scale bar = 100 nm.

Figures 3a–f are the images of the reconstructed amplitude, as cut-off view at the centre of the gold nanocrystal, at times of 0, 2, 4, 6, 8, and 10 hrs of copper diffusion, respectively. It can be seen that after 2 hrs diffusion, the ‘edges' have changed significantly, with the top ‘edge' having disappeared and become an almost flat surface (Fig. 3b). As copper diffuses further into the gold nanocrystal (Fig. 3c–f), the crystalline volume appears to shrink. This is because the BCDI image is derived from the signal of the gold nanocrystal part only, through its (111) Bragg peak. The reconstructions indicate that the copper diffusion removes crystalline material by alloy formation: the affected material either has a different lattice constant or is reoriented. Thus the regions where it is easier for copper atoms to diffuse into become transformed and no longer contribute to the Au (111) diffraction signal during the temporal evolution.

After 4 hrs diffusion, some ‘channels' started to open in the crystal (Fig. 3c). For the same reasons given above, the channels are not likely to be empty, but contain alloy of mis-oriented crystalline material from the conversion of Au to Au-Cu alloy. The number of channels and their length increases with time, up to 40 nm observed after 6 hrs (Fig. 3d) and longer than 100 nm after 8 hrs diffusion (Fig. 3e). Interestingly Fig. 3c–f shows a surface modulation with the crystal surface becoming rougher following diffusion. All these observations suggest that the diffusion is not an isotropic process and the effective differential diffusion rate along different directions results in the formation of channels and rougher surface. The time dependence of the nanocrystal's volume and surface area are discussed further in Supplementary Information 3 where both show roughly linear relationships.

**Figure 4:**
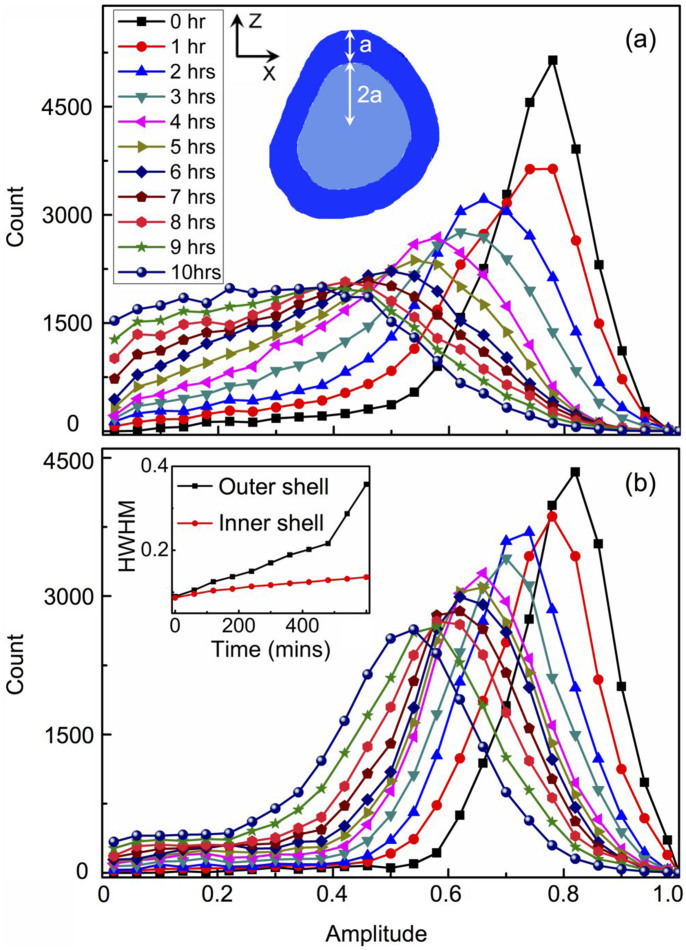
The time dependence of the amplitude histograms for (a) the outer shell and (b) the inner shell of the nanocrystal. The inset of (a) shows the layout of the shells as a XZ plane view. The time dependence of the HWHM of the amplitude histograms for both shells is inset of (b).

To gain more insight into the diffusion process, the gold nanocrystal is defined as a two-shell structure in three dimensions and Fig. 4a inset shows the schematic XZ view of the shells layout as the cut-off image at the centre of the crystal. The thickness ratio of the outer and inner shell is defined to be 1:2 along the radial direction. To access the fraction of crystal remaining in each shell we examine the distribution of reconstructed amplitude values in each shell. Figure 4a shows the amplitude histogram for the outer shell at different diffusion times. For a perfect crystal the amplitude distribution would be a sharp function, but crystal samples have defects and there are Gaussian-distributed errors in the measurements, so it is reasonable that we observe a Gaussian-like distribution. With diffusion proceeding, the peak value of the amplitude histogram decreases and the distribution become broader, after 6 hrs the histogram no longer resembles a Gaussian shape. This continuous increase in the variation of the density is attributed to the deterioration of crystallinity resulted from the diffusion.

The time series of the amplitude histogram for the inner shell (Fig. 4b) shows that although it follows a similar trend as that of the outer shell, it preserves a Gaussian-like distribution after 10 hrs diffusion. The time dependence of the width (half width at half maximum) of the amplitude histograms is extracted and plotted in the inset of Fig. 4b for both shells. The width increases with diffusion time for both shells, and for the inner shell it increases at a slower pace. These results indicate a higher crystallinity and uniformity in the inner shell. This is due to the diffusion starting from the crystal surface, so the deterioration of crystallinity and broadening of the density variation are more substantial in the outer shell. Further analysis of the amplitude histogram yields a diffusion coefficient D of 8.7 × 10^−9^ μm^2^/s, for copper in the gold nanocrystal at 300°C, as described in Supplementary Information 4. It is almost two orders of magnitude larger than that for the bulk sample^50^.

The enhanced diffusion effects have been observed previously from nanocrystal ensembles^29^,^30^,^33^. In-situ TEM was applied to study copper diffusion in gold nanocrystal ensemble with average size of 10 nm at room temperature, and the diffusion coefficient was estimated to be at least 9 orders of magnitude higher than the bulk sample^33^. Other examples including silver in gold nanocrystal ensemble^34^, and gold in semiconductor Ag_2_S nanocrystal ensemble^35^ etc., show diffusion coefficients up to 16 orders of magnitude higher than the bulk^30^. One of the possible reasons for the much higher diffusion coefficient for nanocrystal samples may be due to the higher surface energy. The free energy of activation for atoms to jump from the surface into vacancies inside the nanocrystal is therefore reduced with respect to atoms jumping from lattice sites into vacancies as in bulk diffusion. Here our sample is a single particle with a diameter of 400 nm, the surface to volume ratio is not as high as in the above referenced cases, so the enhancement effect is still significant but not as pronounced. It is worth noting that for the nanocrystal ensembles, the larger free volume in grain boundaries can play a significant role in the diffusion and further complicate the process^30^. In our case one can see from Fig. 3 that on the left side of the nanocrystal, the evolution of the gold lattice seems to be slower than the other sides. This could be due to the grain boundary effect where the measured nanocrystal is in contact with another crystal or the substrate.

## Discussion

Revealing the diffusion processes in a solid on the atomic scale is fundamentally important and difficult. Atomic diffusion in crystalline materials is additionally complicated by the competition of lattice effects which can enhance or impede the process in a directional way. Surface and interface effects can also modify the rates, adding to the complexity of the outcomes for a polycrystalline, heterogeneous or nanostructured material. Our investigation reported here reveals the formation of transient dislocation loops during the diffusion of copper into a single gold nanocrystal, resulted from extra single layer of atoms inserting between the host lattice planes. The results show that it is possible to study the diffusion process on the atomic scale with BCDI. The high spatial resolution of this method allows direct investigation of the size, shape, lattice, surface and interface effects for individual nanocrystal, without having to integrate and average the signals from a large area or ensemble of structures which is necessary in many other methods and can result in ambiguity. Another advantage is that we obtain three-dimensional images for the system under investigation. In the current case, the amplitude images show dynamically how copper invades the gold nanocrystal, starting preferentially along the crystal edges, and the interesting surface modulation due to channel formation along certain directions inside the nanocrystal. With the inherent lattice-distortion sensitivity as demonstrated in this work, and higher brilliance synchrotron sources and free electron lasers planned in the near future, BCDI has the potential to become the method of choice for investigating atomic behaviours in three dimensions for nanomaterials, providing unprecedented insights in many fundamental processes such as diffusion, phase transition, catalysis and imaging phonons (through lattice dynamics^39^) in semiconductors.

## Methods

The sample was prepared by annealing a 20 nm thick gold layer on silicon substrate at 980°C and dewetted to form gold nanocrystals with typical size in the range of 200 nm ~ 1 μm, separated by a few hundred nanometers. The measurements were performed on the Huber diffractometer in experimental hutch 2 of beamline I07, Diamond Light Source. The sample was loaded in the beamline's ultra high vacuum chamber at a base pressure of 5 × 10^−9^ mbar. The X-ray wavelength of 0.133 nm was selected by a Si-(111) double crystal monochromator. A set of JJ slits was used to select a partial coherent beam with a size of 30 μm in horizontal and 40 μm in vertical. The sample holder was resistively heated to 300°C, and the temperature was stable to 0.1°C. When the temperature reached 300°C and stabilized for another 6 hours, the copper evaporation and BCDI measurements were started. A graphite Knudsen-cell thermal evaporator source was heated to 850°C to evaporate copper onto the gold nanocrystal continuously during the measurements. The vapour pressure was 3.0 × 10^−6^ mbar, and the evaporator had a 1 mm aperture at 200 mm from the sample. The total deposited copper is estimated to be around 80 nm thick according to the Hertz-Knudsen equation. A Pilatus 100 k area detector (Dectris, 487 × 195 pixels, 172 × 172 μm^2^ pixel size) was positioned at a distance of 1.0 m from the sample, sufficient for fulfilling the oversampling requirement needed for phasing. In-situ time dependent diffraction patterns were collected by rocking the sample around the gold (111) diffraction, through an angular range of 0.5° in increments of 0.005°, and subsequently stacking the two dimensional patterns to form three dimensional data sets. Three dimensional reconstructions were performed using a guided approach^40^ and partial coherent correction^41^. 10 random starts were initiated with each of them being subjected to 10 iterations of error reduction (ER) fitting followed by 170 iterations of hybrid-input output^49^ (HIO) phasing and then a further 20 iterations of ER. After this first generation, the best fitting was selected and used to generate a further 10 new iterates (Supplementary Information 1). This process was repeated for a total of 6 generations with the final solution averaged from the 2 best fittings.

## Author Contributions

G.X., J.C. and I.R. designed the experiments. G.X. and J.C. prepared the samples. G.X., J.C., I.R., C.N. and J.R. carried out the BCDI measurements. G.X., J.C and I.R. performed data analysis and interpretation. G.X. wrote the manuscript, with contributions and comments from all authors.

## Supplementary Material

Supplementary Information Supplementary Information

Supplementary Information Supplementary Video 1_Temporal diffraction pattern - xz view

Supplementary Information Supplementary Video 2_Reconstructed amplitude after 10hrs diffusion - xz view

Supplementary Information Supplementary Video 3_3D rendering of the Au nanocrystal after 6 hours Cu diffusion
