# Supplementary material for: Atomic Diffusion within Individual Gold Nanocrystal
Source: Sci Rep. 2014 Oct 24;4:6765. doi: 10.1038/srep06765 (PMC4208027; doi:10.1038/srep06765)
Supplement: Supplementary Information [file srep06765-s1.pdf]

Supplementary Information

Atomic Diffusion within Individual Gold Nanocrystal

Gang Xiong[1], Jesse N. Clark[1], Chris Nicklin[2], Jonathan Rawle[2], Ian K. Robinson[1,3]

[1] London Centre for Nanotechnology, University College London, London WC1H 0AH, UK

[2] Diamond Light Source, Harwell Campus, Didcot, OX11 0DE, UK

[3] Research Complex at Harwell (RCaH), Harwell Oxford, Didcot, OX11 0FA, UK

1 Validity of the reconstruction

The oversampling ratios, defined to be the total number of pixels in a direction in the FFT array divided by the number of pixels in the object along the same direction, for the current experiment are $\sigma_x = 2.4$, $\sigma_y = 2.3$, $\sigma_z = 5.1$ respectively. For a reconstruction its error metric, χ^2 is defined as,

$$\chi^2 = \frac{\sum_{j=1}^{J} \left| \sqrt{I^m(j)} - \sqrt{I^k(j)} \right|^2}{\sum_{j=1}^{J} I^m(j)} \qquad (1)$$

where j is the pixel, J the total number of pixels, I^m is the measured intensity and I^k is the estimate of the intensity during the iterative reconstruction process. Error metric represents the degree of match between the measured data and the solution. We performed the reconstruction with 10 different random starts and the best fitting (which have the smallest error metric) from the first generation of iterate was chosen for performing further refinements (see Methods).

Shown in Supplementary Figure S1 is a plot of the error metric (square symbols) of the first generation fittings from 10 different random starts, for the measured diffraction data set after 10 hours diffusion. The low χ^2 values ($<4.8\times10^{-3}$) indicate good agreement between the reconstruction results and the

measured data. Furthermore the quantitative discrepancy was used to directly compare the fittings against each other, which is defined as

$$R_{ij} = \frac{\Sigma|\rho_i - \rho_j|}{\Sigma|\rho_i + \rho_j|} \qquad (2)$$

where ρ_i and ρ_j represent one of the fittings. Figure S1 includes the calculated quantitative discrepancies (triangle symbols) between the solutions from different random starts, with fitting No. 6 as the reference which has the lowest error metric (4.47×10^{-3}). It can be seen that all the quantitative discrepancies are small (with a maximum value of 1.6%) and the fittings are highly consistent. The error metrics and the quantitative discrepancy analysis shown here demonstrate that the reconstruction results are reliable even for the data set measured at the longest diffusion time in the experiment, where the diffraction pattern became non-centrosymetric.

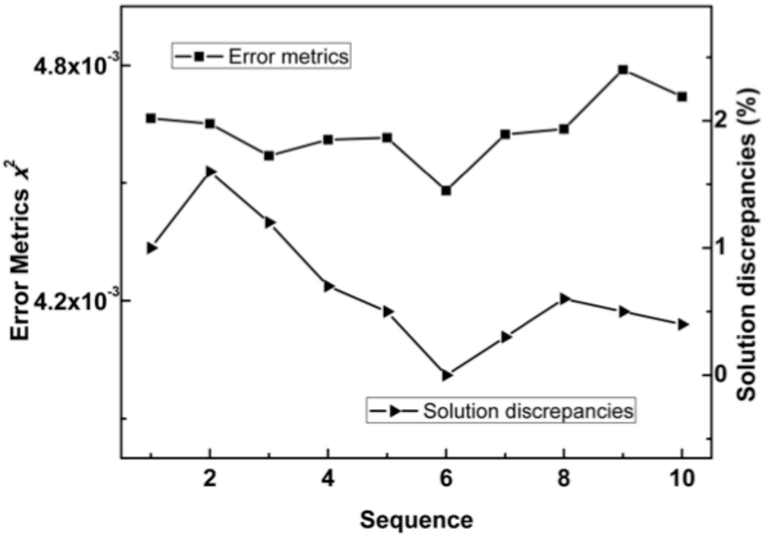

Figure S1. The error metrics of the first generation fittings from 10 different random starts (black squares) and the quantitative discrepancies of the fittings (black squares), for the diffraction data set of the gold nanocrystal measured at 10 hours diffusion time.

It is also worth pointing out that in Fig. 1a-f in the main manuscript we show the zoomed-in images in order to reveal the details of the fringes at the centre part of the diffraction pattern, while the diffraction patterns used for the reconstructions encompassing a nine-times as big area. This is for minimizing the level of the artefacts in the retrieved amplitude, which may incur due to the cut-off of the high frequency signals.

2 Dislocation loop simulation

Simulations were performed to compare with the BCDI measurements. Figure S2 shows the phase structure for a dislocation loop, consisting of two opposite edge dislocations formed from the insertion of a single plane of atoms over a small region between two adjacent lattice planes. The distance between the two edge dislocations is 120 pixels and the Q vector is chosen to be along the horizontal direction. The phase was then modelled using an isotropic elastic theory[1] with the displacement components represented as:

$$u_x = \frac{b}{2\pi}\left(\theta + \frac{\sin 2\theta}{4(1-\nu)}\right) \quad (3)$$

$$\mu_y = -\frac{b}{2\pi}\left(\frac{1-2\nu}{4(1-\nu)} lnr^2 + \frac{cos2\theta}{4(1-\nu)}\right) \quad (4)$$

where r and θ are the polar coordinates centred between the two edge dislocations, and υ the Poisson's constant. The simulated phase is shown in Fig. S2(a), and it presents a dipole-shaped structure with an abrupt change from -π to π. To see how the finite resolution of the imaging system can affect the measurement outcome for the phase structure, a Gaussian filter function (inset of Fig. S2(b)) was further applied to the complex density (includes both the amplitude and the simulated phase) of the dislocation loop structure, with a standard deviation of 64 pixels which is deduced from the finite resolution of the BCDI measurement, and the obtained phase is shown in Fig. S2(b). It can be

seen that after the Gaussian smearing, the full ±π phase wrap and the dipole shape are clearly preserved, consistent with the experiment results.

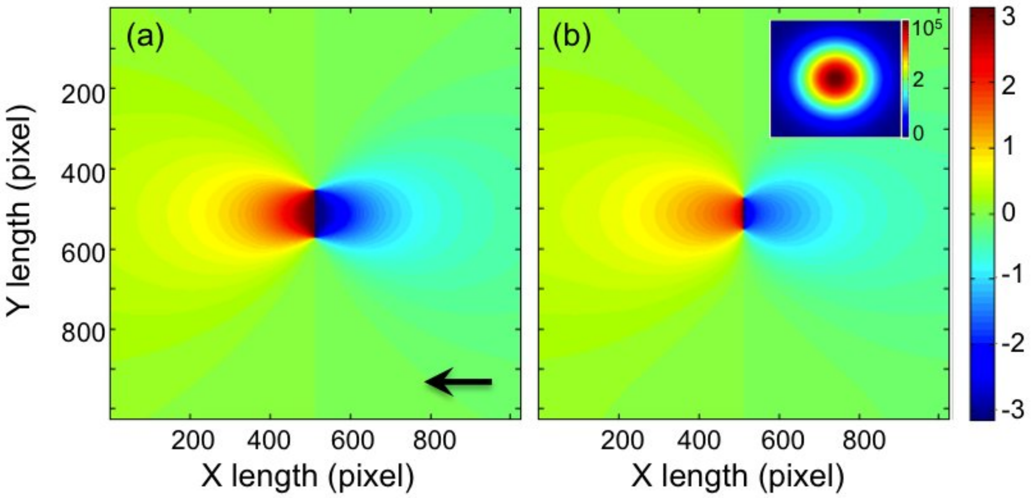

Figure S2. The simulated phase structure from a simple dislocation loop consisting of two opposite edge dislocations (a), and the smeared phase after applied a Gaussian filter (b). Arrow in (a) shows the Q-vector direction. Inset of (b) is the Gaussian filter function applied to the simulated complex density of the dislocation loop.

3 Time dependence of the nanocrystal's volume and surface area

Figure S3 summarises the time dependence of the volume (a) and surface area (b) of the gold nanocrystal. Here the volume and surface are calculated from the reconstructed amplitude at the 40% contour level, and normalized to the values before the copper diffusion, with the error bars showing results between 35% to 45% contour levels. It can be seen that while the volume decreases with time, the surface area increases with time, and both are roughly linear relationships. After 10 hrs of copper diffusion, the crystal volume decreased to 0.68 and the surface area increased to 1.2 of the initial values. The fitted slopes are -5.70×10^{-4} V_0/min for the volume curve and 1.61×10^{-4} S_0/min for the surface curve, as shown in the red lines in Figure S3. Here the V_0 and S_0 are the volume and

surface area of the gold nanocrystal before the copper diffusion, and are 3.6×10^{-20} m^3 and 5.1×10^{-13} m^2 respectively, obtained from the reconstructed amplitude of the nanocrystal. Inserting the values of the V_0 and S_0, the fitted slopes are -3.5×10^{-25} m^3s^{-1} for the volume curve and 1.4×10^{-18} m^2s^{-1} for the surface area curve.

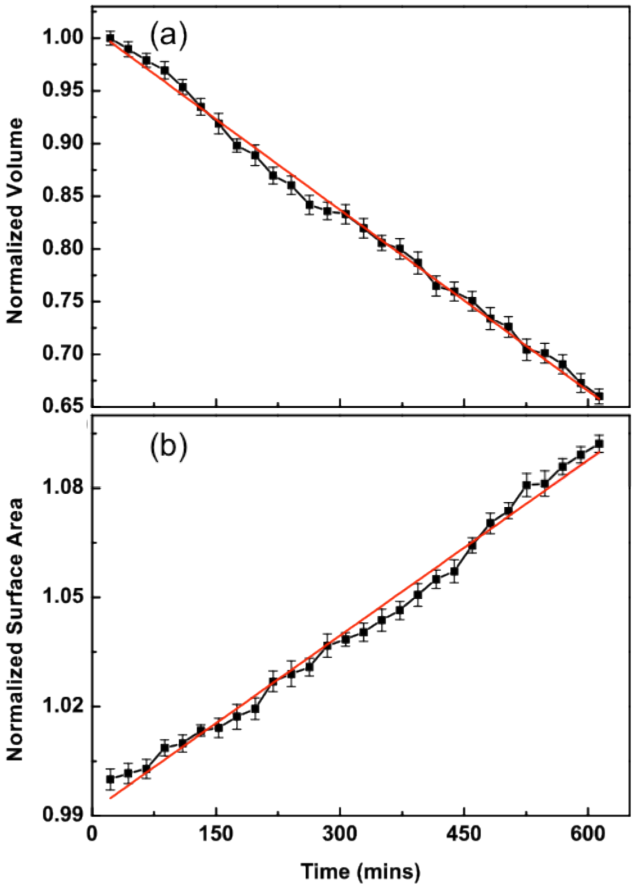

Figure S3. The time dependence of the volume (a) and surface area (b) of the gold nanocrystal, normalized to the initial values before copper diffusion and the linear fit (red lines).

For a sphere, the volume changes with the radius in a relation of $dV = 4\pi\cdot r^2dr$. In the current experiment, as $dV/dt = -3.5\times10^{-25}$ m^3s^{-1}, the diffusion rate along radial direction, $v_r = dr/dt = (-3.5\times10^{-25}/4\pi)\cdot r^{-2}$, scales with r^{-2}, indicating v_r increases rapidly as the crystal shrinks. The accelerating rate of reaction at the

interface between the alloy and the remaining crystal may be due to the higher activity for smaller-sized nanocrystal.

4 Evaluation of the diffusion coefficient

By further analyzing the three dimensional amplitude histogram shown in Fig. 4 of the main manuscript, one can deduce the diffusion coefficient D of copper in the gold nanocrystal. As the diffusion temperature in our experiment is well below $0.3T_m$, where T_m is the melting temperature of gold, the lattice diffusivity is negligible. In this case it is known that a root-mean-square (RMS) relationship[2] can be used for the estimate of the diffusion coefficient D, and is given by Equation (5).

$$x = (Dt)^{1/2} \quad (5)$$

Where t is the time needed to achieve a diffusion distance x. For our experiment, we set an amplitude threshold value of 0.4 and rule that any pixel with a lower value is regarded as having been diffused with copper. One can then estimate the percentage of the gold nanocrystal being converted by the diffusion and work out the average diffusion depth x. Using this method, analysis from the amplitude histogram in Fig. 4 yields a diffusion coefficient D of 8.7×10^{-9} $\mu m^2/s$ for copper in the gold nanocrystal at 300℃. Varying the amplitude threshold value from 0.3 to 0.5 yields a diffusion coefficient D in the range between 4.5×10^{-9} and 1.2×10^{-8} $\mu m^2/s$. Extrapolation from experiment results performed at higher temperatures on bulk gold samples[3] yields a diffusion coefficient D of 9.8×10^{-11} $\mu m^2/s$ for copper in gold at 300℃. The much larger diffusion coefficient obtained from our experiment shows the difference in activity between nanocrystal and bulk material. Here the diffusion coefficient accounts for the overall diffusion effectiveness (thus an average result) of copper inside the single gold nanocrystal, the difference of the diffusion rate along different directions inside the nanocrystal has been smeared out in the modelling.

5 SEM images

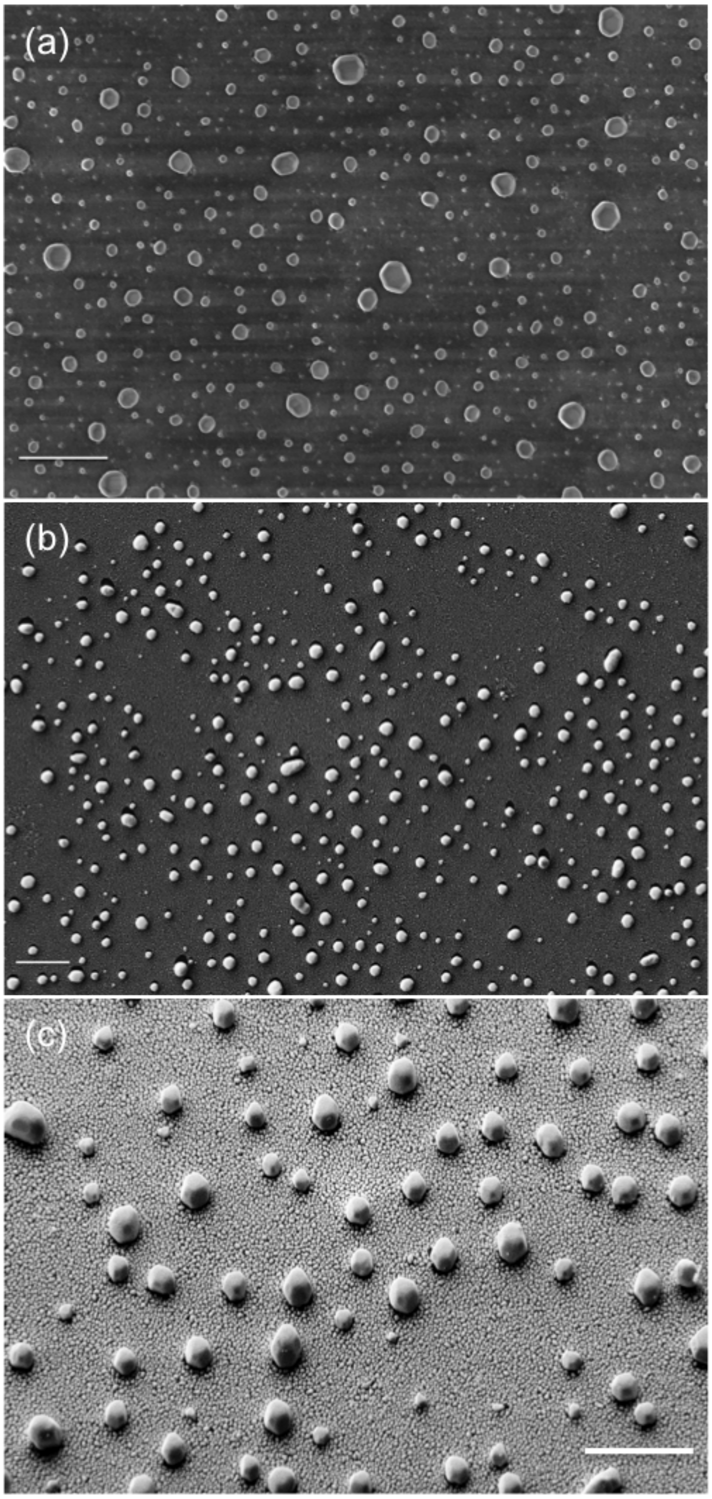

Figure S4. SEM images of the gold nanocrystal sample before (a), and after the diffusion experiments (b) and (c). (c) was imaged at a tilted angle of 45°. Scale bars equal to 2 μm. EDX measurement confirms that the continuous film on the

substrate in (c) consists of copper while for (a) the EDX measurement shows that the corresponding areas consists of Si (the substrate material).

Supplementary Video S1. The temporal evolution of the diffraction pattern at the centre position of the rocking curve for the gold (111) during the copper diffusion, XZ plane view, time interval between successive frames is 22 mins.

Supplementary Video S2. The cross section slices of reconstructed amplitude of the gold nanocrystal at diffusion time of 10 hrs, XZ plane view, shown as a function of the y-coordinate with a distance of 10 nm between successive frames.

Supplementary Video S3. 3D rendering of the density of the gold nanocrystal with the corresponding phase overlaid on the surface, after 6 hours copper diffusion.

References:

1. Hirth, J. P. & Lothe, J. *Theory of Dislocation*. (Wiley, 1982).
2. Mehrer H. *Diffusion in Solids: Fundamentals, Methods, Materials, Diffusion-Controlled Proccesses*. (Springer, 2007).
3. Gale, W. F. & Totemeier, T. C. *Smithells metals reference book*, (Butterworth-Heinemann, 2003).
